# Kollicoat^®^ Smartseal 100P for Developing Theophylline Pellets: Exploring Taste-Masking Potential for Pediatric Applications

**DOI:** 10.3390/pharmaceutics17040413

**Published:** 2025-03-25

**Authors:** Neeraja Komanduri, Mashan Almutairi, Rasha M. Elkanayati, Nagireddy Dumpa, Arun Butreddy, Suresh Bandari, Michael A. Repka

**Affiliations:** 1 Department of Pharmaceutics and Drug Delivery, School of Pharmacy, The University of Mississippi, Oxford, MS 38677, USA; nkomandu@go.olemiss.edu (N.K.); rmelkana@go.olemiss.edu (R.M.E.); ndumpa@go.olemiss.edu (N.D.); abutredd@go.olemiss.edu (A.B.); sbandari@olemiss.edu (S.B.); 2 Department of Pharmaceutics, College of Pharmacy, University of Hail, Hail 81442, Saudi Arabia; m.almutairi@uoh.edu.sa; 3 Pii Center for Pharmaceutical Technology, The University of Mississippi, Oxford, MS 38677, USA

**Keywords:** taste masking, hot-melt extrusion, Kollicoat^®^ Smartseal 100P, pediatric drug delivery, theophylline pellets, Eudragit^®^ EPO

## Abstract

**Background/Objectives:** This study aimed to develop and evaluate taste-masked theophylline pellets using hot-melt extrusion (HME) technology. Additionally, the study evaluates the efficacy of various taste-masking polymers by comparing three pH-dependent polymers, Kollicoat^®^ Smartseal 100P, Eudragit^®^ EPO, and Kollicoat^®^ MAE 100-55, in masking taste and optimizing drug release. **Methods:** Formulations were designed with varying drug loads (10%, 20%, and 30%) and plasticizer concentrations (20% and 30% PEG 1500). Lead formulations were characterized using differential scanning calorimetry (DSC), Fourier transform infrared spectroscopy (FTIR), bitter threshold level, and in vitro release testing. Stability was assessed under accelerated conditions (40 °C ± 2 °C and 75% ± 5% RH) for three months. **Results:** DSC confirmed homogenous dispersion of the drug within the polymer matrix. The optimized formulation comprising 20% theophylline, 20% PEG 1500, and 60% Kollicoat^®^ Smartseal 100P demonstrated effective taste masking, releasing only 1.1% of the drug in simulated salivary fluid (SSF) within two minutes, significantly lower than the pure drug (29.5%, *p* < 0.05), Kollicoat^®^ MAE 100-55 (2.8%, *p* < 0.05), and comparable to Eudragit^®^ EPO (2.1%, *p* > 0.05). Solubility studies further confirmed that theophylline release from the lead formulations remained well below its reported bitter threshold, which could prevent taste perception and mitigate bitterness. In gastric fluid, complete drug release was achieved from Kollicoat^®^ Smartseal 100P and Eudragit^®^ EPO, while Kollicoat^®^ MAE 100-55 exhibited limited release. Stability studies showed that the Kollicoat^®^ Smartseal 100P formulation maintained its texture, taste-masking efficacy, and dissolution profile under accelerated conditions. **Conclusions:** The study demonstrates the novel exploration of Kollicoat^®^ Smartseal 100P for HME application, and its effectiveness in achieving robust taste masking for theophylline, improving patient compliance, particularly in pediatric and geriatric populations.

## 1. Introduction

Oral medications are widely preferred for their convenient administration and high patient acceptance; however, the unpleasant taste of many active pharmaceutical ingredients (APIs) can compromise acceptability, adherence, and ultimately the treatment effectiveness [1]. This challenge is particularly significant for pediatric and geriatric populations since bitter taste and swallowing difficulties often hinder compliance. Thus, the integration of taste-masking strategies is fundamental to the development of oral dosage forms that are effective and patient-centric [2]. A variety of techniques are employed to disguise the bitter taste of APIs in oral formulations [3] including the use of flavoring agents [4], complexation [5], microencapsulation [6], coating [7], ion exchange resin [8], chemical modifications such as prodrugs and salt formation [9], nanotechnology [10], and solid dispersions [11,12].

Among these strategies, hot-melt extrusion has emerged as an advanced technique for taste masking, enabling the creation of solid dispersions for bitter APIs. Furthermore, it supports the integration of complementary taste-masking mechanisms such as complexation [13], ion exchange resin [14], and pH-dependent polymers [15,16] providing combined and highly effective solutions for taste masking. HME involves melting the drug and carrier such as polymer or lipid matrix under heat and mechanical stress to achieve a molecular-level mixing of the blend, forming solid solutions or dispersions. This versatile method is applied not only for taste masking but also for other applications such as solubility enhancement [17,18,19], abuse deterrence [20], and targeted delivery [21]. Additionally, it is utilized to develop various dosage forms, including buccal [22], multi-particulate [23], immediate [24], and extended-release oral formulations [25]. HME offers several advantages over traditional manufacturing methods, such as ease of industry scalability, support for continuous manufacturing, and alignment with FDA objectives for producing high-quality pharmaceutical products [26,27,28,29].

Theophylline (TPL), a bitter-tasting bronchodilator commonly prescribed for pediatric and geriatric patients, serves as a suitable model drug to evaluate taste-masking strategies. Its biological half-life is approximately 4.5 h, and the typical dose ranges from 60 to 200 mg every three to four hours with a maximum recommended daily dose of 600 mg [30]. Its mechanism of action involves smooth muscle relaxation, likely mediated by inhibition of phosphodiesterase PDE III and PDE IV and the suppression of airway responses to stimuli [31]. Despite its therapeutic importance for the prevention and management of breathing difficulties associated with asthma, chronic bronchitis, and other respiratory conditions, its unpleasant taste often poses a barrier to patient compliance. To address this, marketed oral formulations are primarily solutions or elixirs incorporating saccharin sodium and/or sorbitol as sweetening agents, along with fruit, orange, or berry flavors for taste masking. This reflects the common strategy of using artificial sweeteners or high-sugar content to enhance palatability, particularly in pediatric and liquid formulations, which may not always be desirable [32]. As a hydrophilic drug with high aqueous solubility (8.75 mg/mL) [33], a bitter recognition threshold of 0.5 mM (90 µg/mL), and bitter recognition EC50 of 0.6 to 0.9 mM according to BitterDB™ [34], theophylline highlights the critical need for effective taste masking to improve patient adherence and palatability.

Taste-masking can be evaluated using in vivo and in vitro methods, though in vivo techniques are costly and limited by individual variability. In vitro approaches, such as the electronic tongue system, solubility testing against the bitter threshold, and drug dissolution studies in simulated salivary fluid, offer practical and reliable alternatives for assessing taste-masking effectiveness [16].

Polymers with diverse chemical properties are widely used in pharmaceutical applications to mask unpleasant tastes by forming protective barriers that minimize the release of active pharmaceutical ingredients (APIs) in the oral cavity thereby masking bitterness while permitting drug release in specific gastrointestinal tract (GIT) environments. Among these, Eudragit^®^ EPO, a methacrylate-based polymer containing cationic tertiary amino groups, is highly effective for taste masking due to its pH-dependent solubility. The polymer is insoluble at pH above five, preventing drug release in saliva (pH ~6.8) and reducing bitterness perception, while allowing rapid dissolution in the acidic gastric environment for effective drug release. Eudragit EPO has been successfully employed to mask the bitter taste of many APIs, including diclofenac sodium [35], azithromycin [36], and chlorpheniramine maleate [37]. Similarly, kollicoat^®^ Smartseal 100P is a methacrylate-based cationic polymer with a molecular weight of approximately 200,000 Da. This polymer is the spray-dried powder form of Kollicoat^®^ Smartseal 30 D and is characterized by its pH-dependent solubility, remaining insoluble in neutral and basic conditions but dissolving readily at pH levels below 5.5, owing to the protonation of its amino functional groups. Kollicoat^®^ Smartseal 100P has been primarily used as a dispersion for coating applications and has demonstrated effectiveness in masking the unpleasant taste of bitter compounds such as chlorphenamine maleate through fluid-bed coating [38]. However, the polymer is inherently brittle and requires the addition of a plasticizer during processing to improve its mechanical properties and processability [39]. On the other hand, Kollicoat^®^ MAE 100-55 is an anionic copolymer that is insoluble in acidic pH but freely soluble in pH values above 5.5. It is primarily employed as a delayed-release agent, preventing drug release in acidic conditions, and it has also been used for taste masking and moisture protection [40]. However, the use of Kollicoat^®^ Smartseal 100P and Kollicoat^®^ MAE 100-55 in HME processing has been limited, with existing research primarily addressing different grades (Kollicoat^®^ Smartseal 30 D) or alternative applications like abuse deterrence [41], making this study a novel exploration of their applicability in HME-based formulations for taste masking.

One such dosage form that effectively incorporates polymers is the pellet-based formulation. Pellets offer several advantages over conventional unit dosage forms, such as tablets, particularly for pediatric and geriatric patients. These pellets provide dosage flexibility, allowing precise personalization based on the patient’s body weight and serum concentration—an essential feature for theophylline, a drug with a narrow therapeutic window and variable clearance rates. This flexibility is especially critical for pediatric patients, whose clearance rates change significantly with age, and for geriatric patients, who often experience reduced clearance due to age-related physiological changes. Furthermore, pellets can be administered in various ways, including sprinkling on food, blending with liquids, or swallowing directly, making them ideal for patients with swallowing difficulties [30]. Pellets also help minimize inter- and intra-subject variability associated with drugs that are influenced by variable gastric emptying times.

Thus, the primary objective of this study was to develop a palatable and stable oral pellet formulation of theophylline using hot-melt extrusion (HME) to effectively mask its bitter taste. Furthermore, this research seeks to evaluate the taste-masking efficiency and stability of Kollicoat^®^ Smartseal 100P formulations in comparison with Kollicoat^®^ MAE 100-55 and Eudragit^®^ EPO, leveraging their unique polymer properties to identify the most effective taste-masking strategy for theophylline.

## 2. Materials and Methods

### 2.1. Materials

**Figure 1:**
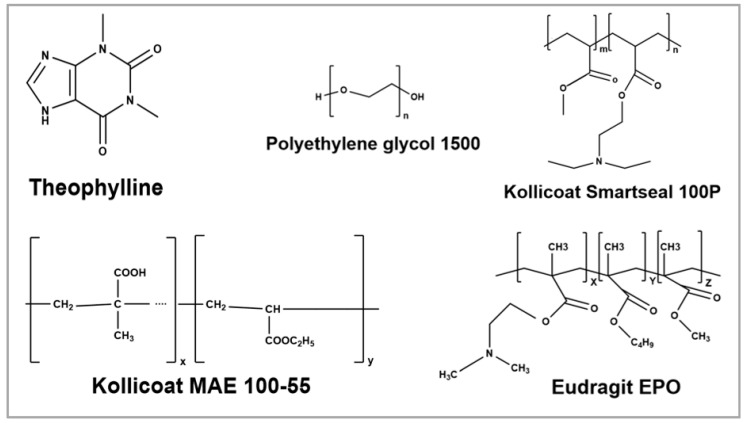
Chemical structure of theophylline and excipients used for formulations (ChemDrawV.23.1.2).

Anhydrous theophylline (TPL) was procured from Sigma Aldrich (Bellefonte, PA, USA); dimethyl aminoethyl methacrylate, butyl methacrylate, and methyl methacrylate, in ratios of 2:1:1 (Eudragit ^®^ EPO) was provided by Evonik (Evonik Industries, Darmstadt, Germany); methyl methacrylate and diethylaminoethyl methacrylate copolymer (Kollicoat^®^ Smartseal 100P) and Methacrylic acid ethyl acrylate copolymer 1:1 (Kollicoat^®^ MAE 100-55) were gifted by BASF (BASF Corporation, Ludwigshafen, Germany); and Polyethylene glycol 1500 (PEG 1500) was acquired from Sigma Aldrich (Bellefonte, PA, USA). Sodium chloride, sodium dihydrogen phosphate monohydrate, disodium hydrogen phosphate heptahydrate, magnesium chloride hexahydrate, and potassium carbonate sesquihydrate were purchased from Fischer Scientific (St. Louis, MO, USA). The chemical structures of the API and the excipients are shown in Figure 1.

### 2.2. Hot-Melt Extrusion Processing

The formulation process consisted of two stages. Stage one involved the optimization of drug load, polymer (Kollicoat^®^ Smartseal 100P), and plasticizer (PEG 1500) concentration. Three different drug loads (10% (*w*/*w*), 20% (*w*/*w*), and 30% (*w*/*w*)) and two plasticizer concentrations (20% *w*/*w* or 30% *w*/*w*) were investigated resulting in six different formulations. Formulations F1 through F6 were extruded, characterized, and evaluated such that the optimized formulation was selected based on drug dissolution studies conducted in simulated salivary fluid and 0.1N HCl. During the second stage of formulation development, the optimized formulation from stage one was compared with two reference formulations, F7 and F8, which incorporated Eudragit^®^ EPO and Kollicoat^®^ MAE 100-55 as polymers, respectively. This comparison aimed to assess the efficiency of Kollicoat^®^ Smartseal 100P against alternative polymers under identical conditions. The compositions and processing parameters of all formulations from both stages are outlined in Table 1.

For physical mixture preparation, the powder blend of TPL, polymer, and PEG 1500 was mixed in a V-shaped blender (GlobePharma, MaxiBlend, New Brunswick, NJ, USA). Each batch was prepared at a size of 30 g. The blends were processed using an 11 mm co-rotating twin-screw extruder (ThermoFisher Scientific, Waltham, MA, USA). A modified screw configuration with a single mixing zone was employed as illustrated in Figure S1. The feeding zone was kept at room temperature, while zones two through the die were maintained at consistent temperatures ranging from 110 °C to 140 °C, adjusted based on the polymer and plasticizer concentrations. Preliminary trials were conducted approximately 30–40 °C above the Tg of the polymers. However, the temperature was further adjusted based on the plasticizer content, as a higher polymer concentration and lower plasticizer content required a higher processing temperature. Throughout the process, torque was closely monitored—lower temperatures resulted in increased torque, while higher temperatures produced excessively soft filaments that were difficult to pelletize. The extrusion process was conducted at a screw speed of 100 rpm with a feed rate of 3–5 g/min. The extrudates produced were cooled at room temperature and pelletized into 2–3 mm pellets using a pelletizer (Type L-001-9482, Thermoscientific, Stone, UK). The pellets were sealed and labeled for further investigation.

### 2.3. Thermogravimetric Analysis (TGA)

Before the extrusion process, the suitability of the HME processing parameters for the API and the excipients was confirmed through a TGA 55 instrument (TA instruments, New Castle, DE, USA). In a platinum pan, approximately 10–20 mg of each ingredient was heated from 25 °C to 400 °C at a constant rate of 10 °C /min under a nitrogen purge. Trios software (version 2.11) was used for data collection and analysis to establish the appropriate processing temperatures for the extrusion process.

### 2.4. Drug Content

Pellets equivalent to 10 mg of theophylline were dissolved in methanol and centrifuged. The supernatant was filtered and the drug content in the formulations was quantified using a UV-visible spectrophotometer at a wavelength of 270 nm using the regression equation derived from the calibration curve of standard solutions. Drug content analysis was performed in triplicate for each formulation, and the results are presented as the mean ± standard deviation. The extraction efficiency of this method was validated by spiking a known amount of the drug into a solvent containing the polymeric mixture yielding a % recovery of >98%.

### 2.5. Differential Scanning Calorimetry (DSC)

A Differential Scanning Calorimeter (Discovery DSC25, TA Instruments, New Castle, DE, USA) was utilized for thermal analysis of the pure API, excipients, physical mixes, and extrudates. At a rate of 10 °C/min, samples were heated from 25 °C to 300 °C. A 50 mL/min flow of ultrapure nitrogen gas was purged to maintain an inert environment. A sample weighing approximately 5 mg was weighed and placed in an aluminum pan with hermetic lid. A reference was made with an empty aluminum pan. Trios software was used to collect and analyze the gathered data (version 2.11).

### 2.6. Fourier Transform Infrared Spectroscopy (FTIR)

FT-IR spectroscopic analysis was performed on the pure drug, excipients, physical mixtures, and the extruded samples to observe the drug–polymer interactions in physical mixtures and extrudates. FT-IR was conducted on a bench FTIR spectrophotometer (Agilent Technologies Cary 660, Santa Clara, CA, USA). The bench was fitted with an ATR accessory (Pike Technologies MIRacle ATR, Madison, WI, USA) that utilized a single-bounce diamond-coated ZnSe internal reflection element. Scanning was conducted over a 600 to 4000 cm^−1^ range at a resolution of 4 cm^−1^.

### 2.7. In Vitro Drug Release in Simulated Salivary Fluid (SSF)

The release of the drug was assessed in SSF to evaluate the taste-masking effectiveness of the formulations. For successful taste masking, the drug release on the tongue has to be minimized [16]. Therefore, the aim of this study is to completely hinder the release of drugs in salivary pH. Drug release profiles of the extruded pellets and free drug equivalents to 200 mg of TPL were tested using a USP apparatus I in 500 mL of SSF. The SSF composition included 0.228 g/L CaCl_2_·2H_2_O, 0.204 g/L Na_2_HPO_4_·7H_2_O, 0.061 g/L MgCl_2_·6H_2_O, 0.603 g/L K_2_CO_3_·1.5H_2_O, 1.017 g/L NaCl, and 0.273 g/L NaH_2_PO_4_·H_2_O, adjusted to a pH of 6.8 to simulate the oral cavity environment [42]. The rotational speed was set to 50 rpm, temperature at 37 °C ± 0.5 °C, and the samples were analyzed every 30 s for up to two minutes with an in-line Rainbow Dynamic Dissolution Monitor^®^ System coupled with Indigo™ software version 4.0 (Pion Inc., Billerica, MA, USA) at wavelength of 270 nm. The experiment was performed in triplicate and the percentage of drug released at the end of two minutes was compared across formulations using ANOVA.

### 2.8. In Vitro Drug Release in 0.1 N HCl

The drug release from the extruded pellets and free drug equivalent to 200 mg of TPL was evaluated using USP apparatus I (Hanson SR8-plusTM; Hanson Research, Chatsworth, CA, USA), in 900 mL of 0.1 N HCl at 37 °C ± 0.5 °C, with a rotational speed of 50 rpm. At predetermined intervals of 5, 10, 15, 30, 45, 60, 90, and 120 min, 2 mL samples were withdrawn and immediately replaced with fresh, preheated medium. The samples were filtered through a 10-μm filter (Quality Lab Accessories LLC, Telford PA, USA), appropriately diluted, and analyzed for drug release using a UV spectrophotometer (Genesys 6; Thermo Scientific, Waltham, MA, USA) at a wavelength of 270 nm.

### 2.9. Bitter Threshold Comparison Study

The solubility of TPL and the extrudates in water was compared to its reported bitter threshold value [43]. To assess solubility, a known quantity of pure drug and extrudates equivalent to 20 mg of TPL were added to separate vials containing distilled water. The mixtures were shaken by hand for 60 s, then centrifuged at 15,000 rpm for 15 min using a Fisher Accuspin Micro 17 (Langenselbold, Germany). The supernatant was diluted with water as needed, and the concentration of TPL was determined as indicated previously.

### 2.10. Stability Studies

Stability testing of the lead formulations was performed using the Caron 6030 Environmental Test Chamber (Caron Products and Services, Marietta, OH, USA) for three months. Formulations were stored in 30 mL glass vials, closed with lids, and placed in the stability chamber under accelerated testing conditions of 40 °C ± 2 °C and 75% ± 5% RH. After three months, drug content analysis was performed to evaluate any potential degradation or loss of the drug during storage. The formulations were also assessed for drug release through in vitro studies in SSF and 0.1N HCl to evaluate their stability. The similarity factor (*f*_2_) for drug release was calculated using the following equation, where an *f*_2_ value greater than 50 indicates similarity between the initial and stored samples [44].

(1) $$f_{2}=50\times log\left\{{\left[1+\left(1∕n\right)\overset{n}{\underset{j=1}{\sum }}{\left|R_{j}-T_{j}\right|}^{2}\right]}^{-0.5}\times 100\right\}$$

where $f_{2}$ = similarity factor used in comparing two dissolution profiles,

*R_j_* = cumulative drug release of initial samples,
*T_j_* = cumulative release of the test sample at predetermined time points,
*n* = number of time points.

### 2.11. Statistical Analysis

The statistical analysis was performed using SPSS (Version 30, IBM SPSS Software Inc., Armonk, NY, USA). A one-way ANOVA was performed to compare the drug release across different formulations in SSF and 0.1 N HCl, and the differences were considered statistically significant at *p* < 0.05 values.

## 3. Results and Discussion

### 3.1. Hot Melt Extrusion Process and Parameters

Initially, a standard screw configuration (three mixing zones) was employed to extrude formulations with Kollicoat^®^ Smartseal 100P. However, extrusion of these formulations was not feasible under this condition due to high torque levels (8–9 N·m) which caused the extruder to shut down. This issue was likely because of the high melt viscosity of the polymer, and the number of mixing zones in the standard screw configuration that are known to increase shear. To address this issue, a modified screw configuration was employed to reduce shear and improve processability. In the modified setup, only one mixing zone with six elements in 60 degrees was retained, instead of three mixing zones from the standard screw configuration. Additionally, PEG 1500 was incorporated at 20% and 30% *w*/*w* to facilitate the extrusion process. To ensure consistency, the same modified screw configuration and PEG 1500 concentrations were employed for the Eudragit^®^ EPO and Kollicoat^®^ MAE 100-55 formulations [35]. This standardized approach with reference formulations allowed for a direct comparison of polymer performance under uniform processing conditions. These adjustments provided sufficient mixing and minimized shear during processing.

Extrusion of stage I formulations (F1 through F6) was carried out at the temperature range of 110 °C to 140 °C. As PEG 1500 concentration and drug load increased, the extrusion temperature was slightly reduced. This reduction was due to the plasticizing effect of PEG 1500, which lowered the melt viscosity of the physical mixture as the polymer concentration decreased. Extrusion of Eudragit^®^ EPO based formulation (F7) was carried out at a temperature of 110 °C, and the Kollicoat^®^ MAE-based formulation (F8) was extruded at the temperature of 130 °C. The obtained extrudates were uniform in color and thickness and the extrudates were subsequently milled into 2–3 mm long pellets using a bench top pelletizer. The formulations, extrusion temperatures, and torque values observed during the process were reported in Table 1.

### 3.2. TGA

**Figure 2:**
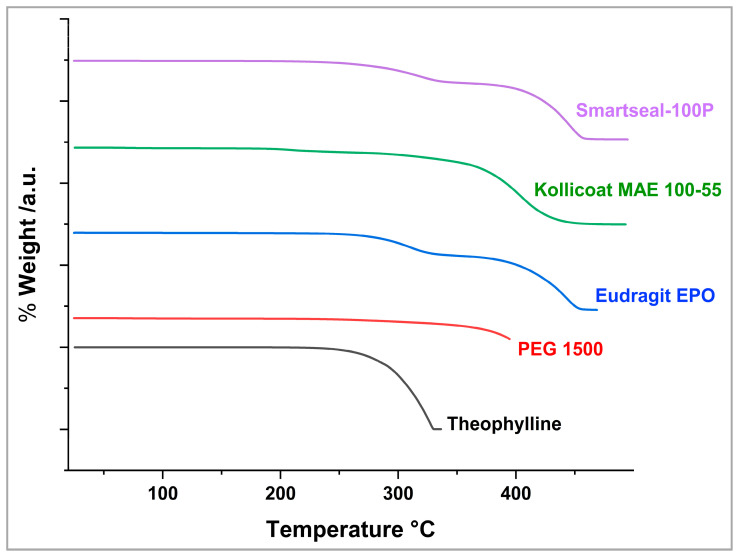
TGA of the drug and excipients.

The thermogravimetric analysis (TGA) was plotted as percentage weight loss against temperature. As shown in Figure 2, TPL began degrading at approximately 242 °C, following a single-stage degradation process, with 50% drug loss recorded at 309.5 °C [45]. Kollicoat^®^ Smartseal-100P exhibited thermal degradation starting at 235 °C, undergoing a two-stage process, with the second stage initiating at 340 °C and 50% degradation being reached at 424 °C. Similarly, Eudragit^®^ EPO degraded in two stages, beginning at 224 °C, with the second stage occurring at 338 °C and 50% weight loss being observed at 417 °C [46]. Kollicoat^®^ MAE 100-55 underwent its first degradation phase at 195 °C and was 50% degraded by 396 °C. Additionally, PEG 1500 displayed a slow degradation pattern, beginning around 256 °C. These findings confirm the thermal stability of TPL and the polymers under processing conditions.

### 3.3. Drug Content Analysis

All pellet formulations showed drug load between 97.1 ± 2.6% and 106.2 ± 1.1% of the theoretical drug load. The small variation in the drug content of the pellets indicated that the filaments produced via HME process achieved a homogenous drug distribution. This finding indicates that the single mixing zone in the modified screw configuration is sufficient to achieve uniform mixing of the drug in the polymer matrix.

### 3.4. DSC Analaysis

**Figure 3:**
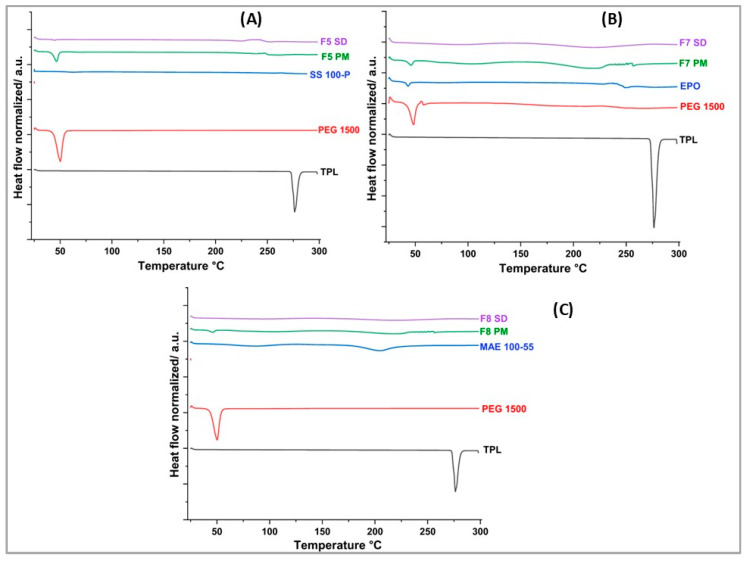
DSC thermograms for theophylline, selected physical mixtures, and formulations from (**A**) F5, (**B**) F7, and (**C**) F8.

DSC was performed to evaluate the physical state of the API in the pure state and after processing. The thermograms of the pure drug, excipients, selected physical mixtures, and extruded formulations are depicted in Figure 3. DSC analysis of the pure API showed a single sharp endothermic peak at 276 °C, with an onset temperature of 274 °C, and an enthalpy of 157.9 J/g, which corresponds to its melting point. These findings are in accordance with the values reported in the literature [47]. This sharp endotherm of the drug at 276 °C was not seen in all the extruded formulations indicating that the drug was successfully dispersed within the polymer matrix during the extrusion process. However, the melting peak was not observed in F5 PM, making it difficult to confirm complete amorphization of the drug in the solid dispersions. Nevertheless, it is important to note that the amorphization of the drug within the polymer was not a primary objective of this study, as the drug already possesses high solubility. Instead, the primary goal was to achieve uniform dispersion of the drug within the polymer to improve its taste masking, rather than to enhance solubility.

### 3.5. FTIR Analysis

**Figure 4:**
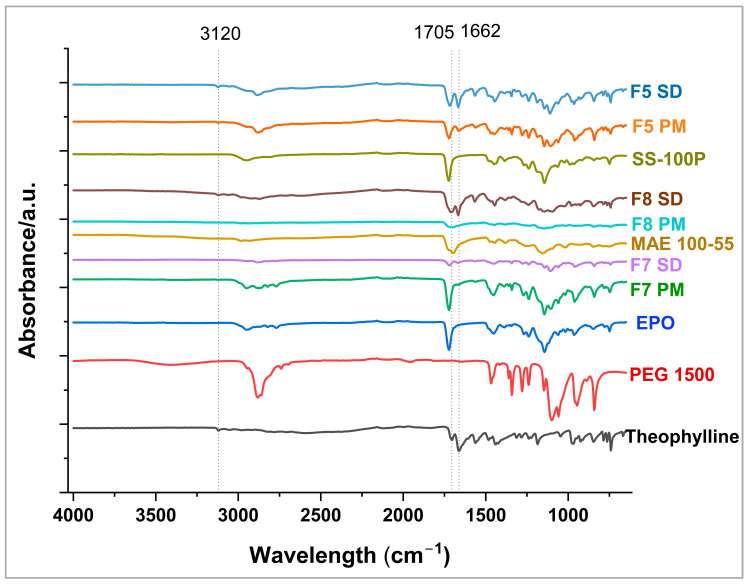
FTIR overlay of pure drug, selected physical mixture, and formulations.

FTIR was performed to identify the intermolecular interactions between the drug and polymers. The FTIR spectra for the formulations are shown in Figure 4. The characteristic bands of the drug and the pure polymers are displayed in Table 2. A key observation from the spectra is the retention of the characteristic N-H stretching vibration of theophylline observed at 3120 cm^−1^ in the physical mixtures, whereas this band is completely absent in the solid dispersions of F7 and F8. Another finding is the reduction in band intensity which is particularly significant for F7 SD where the spectra appear to be diminished compared to the F7 PM. Additionally, the carboxylic C=O stretching vibration of theophylline observed at 1662 cm^−1^ appears to be intensified in F5 and F8 solid dispersion compared to the physical mixtures. The above observations suggest potential hydrogen bond formation between the N-H group of drug and carbonyl groups in the polymers, and possibly the carbonyl group of the drug with hydroxyl group of the polymers. These interactions likely contribute to improved molecular dispersion of the drug within the polymer matrix, potentially enhancing its stability and taste-masking properties.

### 3.6. Evaluation of Taste Making Effectiveness and Drug Release in SSF

Taste perception is highly subjective, varying significantly between individuals, which necessitates precise experimental setups for accurately measuring taste thresholds. Both in vivo and in vitro test methods are employed to assess the taste of pharmaceutical formulations. In vivo taste evaluations involve applying formulations to the tongues of human subjects who assign numeric values to bitterness levels. However, this method is costly, subject to ethical concerns, and is prone to variability [50]. On the other hand, in vitro methods, such as the electronic tongue system, are still used, although primarily in the food industry. This system uses electronic sensors that are analogous to the human gustatory system. Upon contact with a bitter molecule, the sensor generates a change in electric potential which is proportional to the bitterness of the molecule [51]. However, this method comes with several challenges as interactions between formulation ingredients can lead to a potential misinterpretation of the results, and sensor responses vary based on the ionization state of molecules [52]. This is particularly problematic with TPL, since previous research highlighted the challenges of assessing the bitterness of TPL using the multichannel taste sensor (402B) with sensor membranes. TPL is a neutral molecule that does not dissociate to generate a considerable change in electric potential of negatively charged membranes, despite exhibiting significant bitterness in human gustatory tests [53].

Another in vitro approach involves measuring the drug dissolution of formulations in simulated salivary fluid. This is an essential tool for measuring the reduction in release of the API in the oral cavity, which implies less interaction of the drug with the tase buds. The time required for conducting the test and evaluating the taste-masking effectiveness can last from a few seconds to several minutes, but should not exceed five minutes, as it is unrealistic for a dosage form to remain in the mouth for such a duration. Previous studies have conducted dissolution tests for two minutes, including assessing the effectiveness of Eudragit EPO in masking the bitterness of ketoprofen [54] and gelatin in masking the taste of acetaminophen and ibuprofen [55].

**Figure 5:**
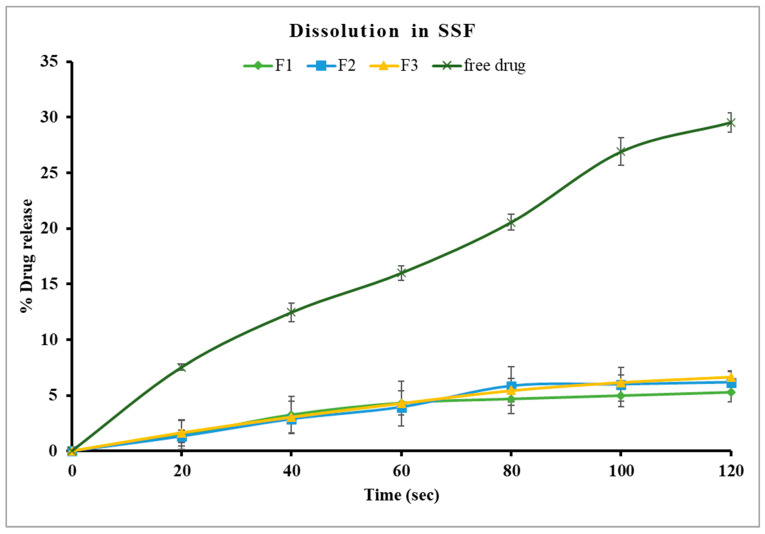
Drug dissolution of theophylline and Kollicoat Smartseal 100P based formulations containing 30% PEG 1500 (F1, F2, F3) in SSF, pH 6.8.

In this study, formulations F1 through F3, containing 30% PEG 1500 with drug loads of 10%, 20%, and 30% *w*/*w*, respectively, were evaluated using in vitro dissolution in SSF. The samples were analyzed every 20 s for up to two minutes with an in-line Rainbow Dynamic Dissolution Monitor^®^ System which enabled real time data collection without interrupting the dissolution process. The results showed that F1 released 5.3%, F2 released 6.2%, and F3 released 6.7% of the drug within two minutes. These values were significantly lower (*p* < 0.05) compared to the free drug, which exhibited a release of around 30% within the same time interval, as illustrated in Figure 5.

**Figure 6:**
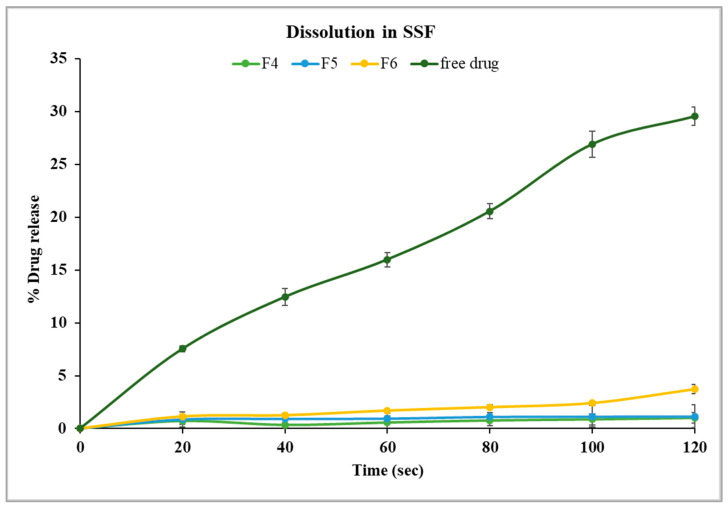
Drug dissolution of theophylline- and Kollicoat^®^ Smartseal 100P-based formulations containing 20% PEG 1500 (F4, F5, F6) in SSF, pH 6.8.

Alternatively, formulations F4, F5, and F6, which contained a lower concentration of PEG 1500 (20% *w*/*w*), had better control of drug release in comparison to formulations with 30% *w*/*w* PEG 1500. Specifically, F4 and F5 showed only 1.1% drug release after two minutes, while F6 exhibited a significantly higher release of 3.7% (*p* < 0.05) under identical conditions, as shown in Figure 6. These findings suggest that an increase in PEG 1500 concentration enhanced the drug dissolution in the release media due to its hydrophilic nature, which likely improved the drug solubility [41]. Overall, stage I formulations showed significantly lower (*p* < 0.05) drug release in SSF compared to the free drug. F4 and F5 were highly effective in limiting the drug release in the buccal media, thereby masking the bitterness of TPL. The formulation F5 was selected for further studies as it has higher drug load, which may reduce the total weight of pellets required for dosing.

**Figure 7:**
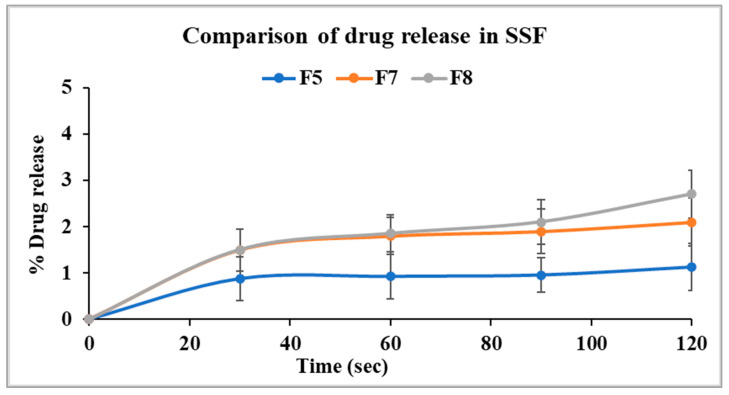
Comparison of drug dissolution from selected formulations F5, F7, and F8 in SSF.

The release profile of the optimized formulation from stage I (F5) was compared with F7 and F8 to evaluate the taste-masking efficiency of the Kollicoat^®^ polymer grades (SS-100P and MAE 100-55) relative to Eudragit^®^ EPO. As illustrated in Figure 7, F5 showed 1.1% drug release in two minutes, while F7 showed a slightly higher release of 2.1% under similar conditions. Statistical analysis revealed no significant difference in the release profiles of F5 and F7 after two minutes (*p* > 0.05). This confirms that the taste-masking efficiency of Kollicoat^®^ Smartseal 100P is comparable to Eudragit^®^ EPO. In contrast, Kollicoat^®^ MAE 100-55 demonstrated a drug release of only 2.8% in the first two minutes despite its high solubility in salivary pH as compared to Kollicoat^®^ Smartseal 100P and Eudragit^®^ EPO. However, ANOVA analysis showed that the percentage of drug released from F8 was significantly higher than that of F5 (*p* < 0.05). It is worth noting that the release study was conducted in 500 mL of buccal media, which significantly exceeds the estimated physiological volume of fluid in the buccal cavity. Consequently, it is anticipated that the release profile may be more favorable under in vivo conditions. Further in vivo studies are necessary to validate and corroborate these findings.

### 3.7. In Vitro Drug Release in 0.1 N HCl

The in vitro release studies in 0.1 N HCl were conducted for all formulations from both stages against the free drug to confirm the complete dissolution of the drug from the polymer matrices under acidic conditions. The free drug exhibited 100% drug within 60 min. In comparison, F1, F2, F3, and F4 formulations achieved complete drug release within 45 min, while F5 and F6 formulations required 90 and 120 min, respectively (Figures S2 and S3). Interestingly, F6 exhibited an initial rapid release, with more than 80% of the drug dissolving in 30 min, followed by a slower but complete dissolution within two hours. This decline in dissolution rate could be due to recrystallization of amorphous TPL upon exposure to the dissolution medium, a phenomenon that is commonly observed with amorphous solid dispersions [56].

Formulations containing 30% PEG 1500 showed slightly higher drug release as compared to those with 20% PEG 1500 at all three drug load levels. This faster release with higher PEG 1500 in both 0.1N HCL and pH 6.8 media is attributed to the increased solubility and pore forming ability of PEG 1500 [24].

**Figure 8:**
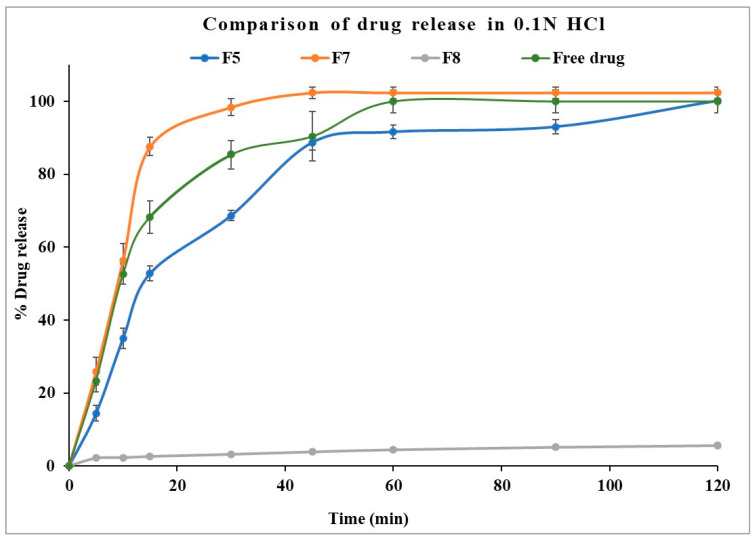
Comparison of drug dissolution from selected formulations F5, F7, and F8 in 0.1 N HCl.

The dissolution profile of the optimized formulation (F5) and stage II formulations (F7 and F8) in gastric pH medium are displayed in Figure 8. Formulation with Eudragit^®^ EPO displayed faster drug dissolution in acidic conditions comparable to the free drug. Eudragit^®^ EPO is highly soluble in acidic pH (<5), contributing to its effective drug release. Kollicoat^®^ Smartseal 100P released approximately 68% of the drug within 30 min, while Eudragit^®^ EPO achieved nearly 100% drug release within the same time frame. The faster release from Eudragit^®^ EPO compared to Kollicoat^®^ Smartseal 100P can be attributed to its greater hydrophilicity, which is enhanced by the dimethyl group in its structure, as opposed to the diethyl group in Kollicoat^®^ Smartseal 100P. In contrast, Kollicoat^®^ MAE exhibited only about 6% drug release over two hours, reflecting its high insolubility in acidic pH, which explains its limited release under these conditions.

### 3.8. Bitter Threshold Comparison

Upon shaking the pure drug in water for one minute, the solubility of TPL was found to be 1450 µg/mL, which is greater than its bitter threshold value of 108–162 µg/mL, as reported in BitterDBTM [34]. This high solubility of TPL in water implies that TPL can easily come in contact with the taste buds and produce a bitter response. To effectively mask the bitter taste, it is essential to control the drug’s solubility in water such that the amount of the drug released in the oral cavity remains below the bitter threshold value.

The optimized formulations demonstrated significantly lower solubility values compared to the free drug in water. The drug solubility values were measured at 37.8 ± 9.6 µg/mL, 27.3 ± 0.9 µg/mL, and 45.9 ± 19.2 µg/mL for Eudragit^®^ EPO (F7), Kollicoat^®^ Smartseal 100P (F5), and Kollicoat^®^ MAE 100-55(F8) formulations, respectively. Thus, the solubility values of theophylline from all three formulations ranged from 27 to 46 µg/mL which are well below the bitter threshold of TPL. Moreover, statistical analysis revealed no significant difference among the solubility values of the formulations (*p* > 0.05), indicating that all the tested polymers effectively suppressed drug release in water. This suppression prevents drug release above the threshold level, thereby mitigating the potential for a bitter taste. The results align with the findings from the SSF dissolution studies which further confirm the taste-masking ability of the tested polymers.

### 3.9. Stability Study 

Based on the findings from the in vitro release study in both media and the bitter threshold comparative results, formulations developed with Kollicoat^®^ Smartseal 100P (F5) and Eudragit EPO (F7) were selected for stability evaluation under accelerated testing conditions for three months. Whereas, F8 was excluded from this experiment, despite its taste-masking efficiency, due to its different drug dissolution behavior in SGF media.

**Figure 9:**
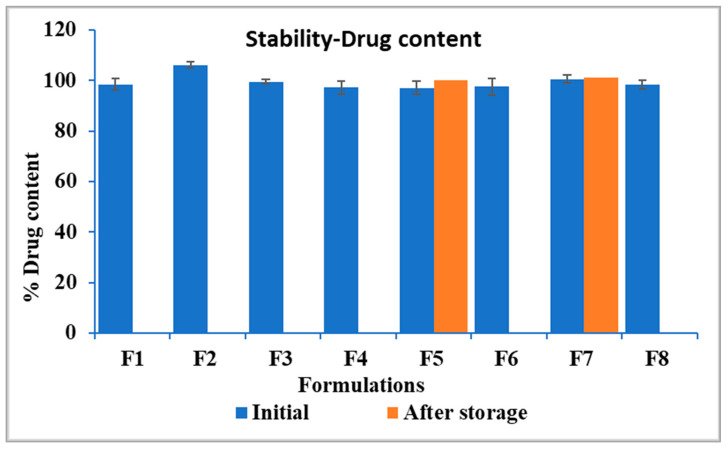
Drug content of the formulations, F5 and F7 showing results before and after storage.

**Figure 10:**
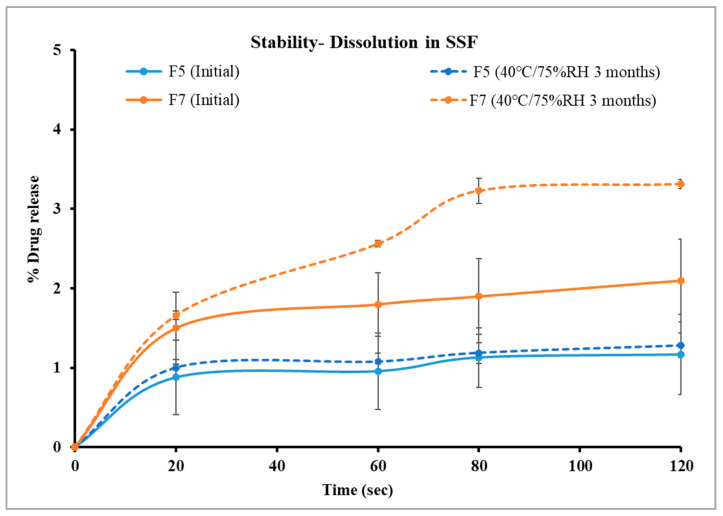
Comparison of drug dissolution from selected formulations F5 and F7 in SSF after storage.

After three months of storage, the drug content of the formulations was stable, as indicated in Figure 9. As illustrated in Figure 10, the drug release profile of F5 in SSF demonstrated 1.28% drug release in two minutes, compared to its initial release of 1.17% on day 0. Similarly, F7 exhibited 3.31% drug release in two minutes, compared to its initial results of 2.1%. Both formulations showed comparable results before and after storage as indicated by similarity factors for F5 and F7, respectively (*f*_2_ = 99.9, 78.65). These findings suggest that the formulations maintained their integrity and effectively retained their taste-masking ability after three months of accelerated stability testing. However, the dissolution profile of F7 in SSF after storage was significantly higher than that of F5, as demonstrated with statistical analysis using an independent t-test that compared drug released from both formulations after two minutes (*p* < 0.05). This faster drug release of F7 is attributed to increased molecular mobility of the polymer matrix at elevated storage conditions. It was reported in the previous literature that even small amounts of absorbed moisture lower Eudragit EPO Tg and induce a plasticizing effect, making it more flexible and potentially enhancing drug diffusion. This plasticization effect likely contributed to the increased drug release rate after storage under high-temperature and humidity conditions [57].

**Figure 11:**
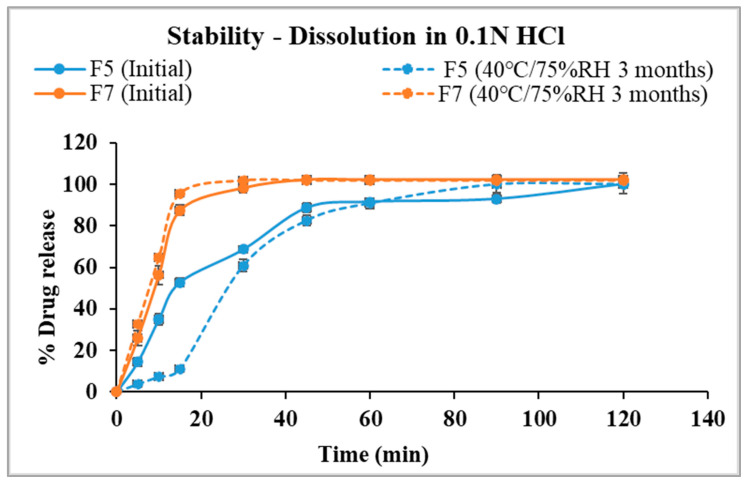
Comparison of drug dissolution from selected formulations F5 and F7 in 0.1 N HCl after storage.

The in vitro dissolution profiles in 0.1 N HCl after three months of storage are demonstrated in Figure 11. F5 achieved complete drug release in 90 min, though its dissolution profile showed a slower release in the first 30 min after storage compared to the initial formulation. Specifically, 61% of the drug was released within 30 min post-storage, compared to 68.7% before storage. Notably, the release tends to be faster from the stored formulation after 30 min. Although the percentage released from both conditions is the same in 120 min, additional long-term stability studies in an acidic medium will be necessary to monitor and ensure consistency in the release profile.

F7 achieved complete drug release in 45 min and had consistent release profiles in acidic media for initial and after storage results. Changes in the physical texture of F7 were observed after storage, with the pellets sticking to the glass vials [58]. The observation aligns with the significantly increased release rate in the SSF media and the slight increase in the gastric media.

Overall, the stability studies demonstrated that F5 maintained its integrity, drug content, and taste-masking effectiveness under accelerated stability testing conditions for up to three months. Its consistent release profile, combined with its ability to retain taste-masking properties, highlights F5 as a stable and effective formulation. However, further investigation is needed to ensure its suitability for pediatric use. While PEG is generally accepted at 136 gm/day for pediatrics according to the Safety and Toxicity of Excipients for Pediatrics (STEP) database, Kollicoat^®^ Smartseal 100P has not been explored for this population. Thus, this study represents an early phase of pre-formulation development, highlighting the need for additional preclinical studies, including juvenile toxicity assessments, pharmacokinetic evaluations, and long-term stability studies to establish its safety and efficacy in pediatric patients [59]. Furthermore, the ICH S11 guideline emphasizes the necessity of conducting juvenile animal toxicity studies when safety data in pediatric populations is insufficient [60]. Additionally, clinical trials (Phases 1–3) will also be necessary to evaluate pharmacokinetics, pharmacodynamics, and therapeutic efficacy in children.

## 4. Conclusions

The study demonstrated the development and optimization of Kollicoat^®^ Smartseal 100P-based pellets for masking the bitter taste of theophylline. Various drug loads and plasticizer concentrations were incorporated for optimization of release in simulated salivary and gastric fluids. DSC analysis suggested the dispersion of theophylline within the polymer matrix, while FTIR studies indicated molecular interactions between the drug and excipients. The optimized formulation was compared to reference formulations developed with Eudragit^®^ EPO and Kollicoat^®^ MAE 100-55, demonstrating comparable efficacy in suppressing drug release at salivary pH, validating its effectiveness as a taste-masking polymer. In vitro dissolution testing revealed that higher plasticizer concentrations improved drug solubility in 0.1 N HCl but compromised taste-masking performance, particularly in SSF media. Kollicoat^®^ MAE showed effective taste-masking properties, yet the drug release was limited to 6% in gastric media after two hours. Stability studies under accelerated conditions confirmed that the physical texture, taste-masking ability, and dissolution profiles of the Kollicoat^®^ Smartseal 100P-based formulation were retained for up to three months. In conclusion, Kollicoat^®^ Smartseal 100P is an effective polymer for taste masking and formulating stable pellets via HME. This study highlights its potential as a promising alternative for the taste masking of bitter drugs, particularly in pediatric formulations to enhance acceptability and compliance.

## Figures and Tables

**Table 1:** Formulation and extrusion parameters of theophylline formulations.

Stage	No.	TPL	PEG 1500	Polymer	Polymer Name	Temperature	Screw Speed	Torque
		(% *w*/*w*)				(°C)	RPM	N·m
Stage I	F1	10	30	60	Kollicoat^®^ Smartseal 100P (SS 100P)	130	100	1
	F2	20	30	50		120	100	1
	F3	30	30	40		110	100	1
	F4	10	20	70		140	100	1
	F5	20	20	60		130	100	1
	F6	30	20	50		130	100	2
Stage II	F7	20	20	60	Eudragit^®^ EPO	110	100	1
	F8	20	20	60	Kollicoat^®^ MAE 100-55	130	100	2

**Table 2:** FTIR spectra of the pure drug and polymers.

Ingredient	Description	Wavelength cm^−1^	References
Theophylline	N-H stretching vibration C-H aliphatic and aromatic stretching vibrations C=O stretching vibration N-H bending vibration	3120 3047, 2986, 2918 1705, 1657 1559	[48]
PEG 1500	C-H stretching vibration C-H bending C-O-C stretching vibration	2877 1467 1059	[24]
Kollicoat^®^ MAE 100-55	-OH stretching vibration Ester C=O and carboxyl group stretching vibration C-O-C ester stretching C-H stretching vibration	3528 1725, 1698 1151 2991, 2926	
Eudragit EPO	Dimethyl amino group N-CH_3_ stretching vibration C=O stretching vibration C-H bending C-O-C stretching	2950 1725 1450 1139	[49]
Kollicoat^®^ Smartseal 100-P	Tertiary amine	2950	
	C-H aliphatic and aromatic stretching vibrations	2805	
	Ester C=O stretching vibration	1725	
	C-O stretching	1143	

## Data Availability

The data presented in this study are available within the article.

## Supplementary Materials

The following supporting information can be downloaded at: https://www.mdpi.com/article/10.3390/pharmaceutics17040413/s1, Figure S1: Screw configuration employed for extruding the formulations; Figure S2: Drug dissolution of theophylline and Kollicoat Smartseal 100P based formulations containing 30% PEG 1500 (F1, F2, F3) in 0.1 N HCl; Figure S3: Drug dissolution of theophylline and Kollicoat Smartseal 100P based formulations containing 20% PEG 1500 (F4, F5, F6) in 0.1 N HCl.

## References

[1] Hauber B., Hand M.V., Hancock B.C., Zarrella J., Harding L., Ogden-Barker M., Antipas A.S., Watt S.J. (2024). Patient Acceptability and Preferences for Solid Oral Dosage Form Drug Product Attributes: A Scoping Review. Patient Prefer. Adherence.

[2] Steiner D., Meyer A., Immohr L.I., Pein-Hackelbusch M. (2024). Critical View on the Qualification of Electronic Tongues Regarding Their Performance in the Development of Peroral Drug Formulations with Bitter Ingredients. Pharmaceutics.

[3] Sanjay L.R., Ashokbhai M.K., Ghatole S., Roy S., Kashinath K.P., Kaity S. (2025). Strategies for Beating the Bitter Taste of pharmaceutical formulations towards better therapeutic outcomes. RSC Pharm..

[4] Ahmed K.K., Kassab H.J., Al Ramahi I.J., Alwan Z.S. (2023). Taste Masking of Steroids for Oral Formulations. Turk. J. Pharm. Sci..

[5] Liu T., Wan X., Luo Z., Liu C., Quan P., Cun D., Fang L. (2019). A Donepezil/Cyclodextrin Complexation Orodispersible Film: Effect of Cyclodextrin on Taste-Masking Based on Dynamic Process and in Vivo Drug Absorption. Asian J. Pharm. Sci..

[6] Al-kasmi B., Alsirawan M.B., Bashimam M., El-zein H. (2017). Mechanical Microencapsulation: The Best Technique in Taste Masking for the Manufacturing Scale—Effect of Polymer Encapsulation on Drug Targeting. J. Control. Release.

[7] Chiarugi I., Biagi D., Nencioni P., Maestrelli F., Valleri M., Mura P.A. (2024). Taste Masking of Dexketoprofen Trometamol Orally Disintegrating Granules by High-Shear Coating with Glyceryl Distearate. Pharmaceutics.

[8] Siddiqui F., Shoaib M.H., Ahmed F.R., Qazi F., Yousuf R.I., Usmani M.T., Saleem M.T., Ahmed K. (2023). Formulation Development and Optimization of Taste-Masked Azithromycin Oral Suspension with Ion Exchange Resins: Bioanalytical Method Development and Validation, in Vivo Bioequivalence Study, and in-Silico PBPK Modeling for the Paediatric Population. J. Drug Deliv. Sci. Technol..

[9] Mishra A., Upadhyay P.K., Niranjan A.K. (2022). Formulation and Optimization of Anti-Epileptic Drug Delivery System for Fast Dissolving Intraoral Drug. World J. Pharm. Res..

[10] Krieser K., Emanuelli J., Daudt R.M., Bilatto S., Willig J.B., Guterres S.S., Pohlmann A.R., Buffon A., Correa D.S., Külkamp-Guerreiro I.C. (2020). Taste-Masked Nanoparticles Containing Saquinavir for Pediatric Oral Administration. Mater. Sci. Eng. C.

[11] Gryczke A., Schminke S., Maniruzzaman M., Beck J., Douroumis D. (2011). Development and Evaluation of Orally Disintegrating Tablets (ODTs) Containing Ibuprofen Granules Prepared by Hot Melt Extrusion. Colloids Surf. B Biointerfaces.

[12] Patil P.S., Suryawanshi S.J., Patil S.S., Pawar A.P. (2024). HME-Assisted Formulation of Taste-Masked Dispersible Tablets of Cefpodoxime Proxetil and Roxithromycin. J. Taibah Univ. Med. Sci..

[13] Malaquias L.F.B., Sá-Barreto L.C.L., Freire D.O., Silva I.C.R., Karan K., Durig T., Lima E.M., Marreto R.N., Gelfuso G.M., Gratieri T. (2018). Taste Masking and Rheology Improvement of Drug Complexed with Beta-Cyclodextrin and Hydroxypropyl-β-Cyclodextrin by Hot-Melt Extrusion. Carbohydr. Polym..

[14] Tan D.C.T., Ong J.J., Gokhale R., Heng P.W.S. (2018). Hot Melt Extrusion of Ion-Exchange Resin for Taste Masking. Int. J. Pharm..

[15] Wang H., Dumpa N., Bandari S., Durig T., Repka M.A. (2020). Fabrication of Taste-Masked Donut-Shaped Tablets Via Fused Filament Fabrication 3D Printing Paired with Hot-Melt Extrusion Techniques. AAPS PharmSciTech.

[16] Maniruzzaman M., Boateng J.S., Chowdhry B.Z., Snowden M.J., Douroumis D. (2014). A Review on the Taste Masking of Bitter APIs: Hot-Melt Extrusion (HME) Evaluation. Drug Dev. Ind. Pharm..

[17] Sawant K., Elkanayati R.M., Almotairy A., Repka M.A., Almutairi M. (2025). Clotrimazole Mucoadhesive Films with Extended-Release Properties for Vaginal Candidiasis—A Hot-Melt Extrusion Application. J. Pharm. Sci..

[18] Elkanayati R.M., Omari S., Youssef A.A.A., Almutairi M., Almotairy A., Repka M., Ashour E.A. (2024). Multilevel Categoric Factorial Design for Optimization of Raloxifene Hydrochloride Solid Dispersion in PVP K30 by Hot-Melt Extrusion Technology. J. Drug Deliv. Sci. Technol..

[19] Elkanayati R.M., Karnik I., Uttreja P., Narala N., Vemula S.K., Karry K., Repka M.A. (2024). Twin Screw Melt Granulation of Simvastatin: Drug Solubility and Dissolution Rate Enhancement Using Polymer Blends. Pharmaceutics.

[20] Palekar S., Nukala P.K., Patel K. (2022). Aversion Liquid-Filled Drug Releasing Capsule (3D-RECAL): A Novel Technology for the Development of Immediate Release Abuse Deterrent Formulations Using a Fused Deposition Modelling (FDM) 3D Printer. Int. J. Pharm..

[21] Alshammari N.D., Almotairy A., Almutairi M., Zhang P., Al Shawakri E., Vemula S.K., Repka M.A. (2024). Colon-Targeted 3D-Printed Mesalamine Tablets: Core-Shell Design and in Vitro/Ex-Vivo Evaluation. J. Drug Deliv. Sci. Technol..

[22] Elkanayati R.M., Chambliss W.G., Omari S., Almutairi M., Repka M.A., Ashour E.A. (2022). Mucoadhesive Buccal Films for Treatment of Xerostomia Prepared by Coupling HME and 3D Printing Technologies. J. Drug Deliv. Sci. Technol..

[23] Vemula S.K., Narala S., Uttreja P., Narala N., Daravath B., Kalla C.S.A., Baisa S., Munnangi S.R., Chella N., Repka M.A. (2024). Quality by Design (QbD) Approach to Develop Colon-Specific Ketoprofen Hot-Melt Extruded Pellets: Impact of Eudragit^®^ S 100 Coating on the In Vitro Drug Release. Pharmaceutics.

[24] Omari S., Ashour E.A., Elkanayati R., Alyahya M., Almutairi M., Repka M.A. (2022). Formulation Development of Loratadine Immediate-Release Tablets Using Hot-Melt Extrusion and 3D Printing Technology. J. Drug Deliv. Sci. Technol..

[25] Elkanayati R.M., Darwesh A.Y., Taha I., Wang H., Uttreja P., Vemula S.K., Chambliss W.G., Repka M.A. (2024). Quality by Design Approach for Fabrication of Extended-Release Buccal Films for Xerostomia Employing Hot-Melt Extrusion Technology. Eur. J. Pharm. Biopharm..

[26] Almutairi M., Hefnawy A., Almotairy A., Alobaida A., Alyahya M., Althobaiti A., Adel Ali Youssef A., Elkanayati R.M., Ashour E.A., Smyth H.D.C. (2024). Formulation and Evaluation of Inhaled Sildenafil-Loaded PLGA Microparticles for Treatment of Pulmonary Arterial Hypertension (PAH): A Novel High Drug Loaded Formulation and Scalable Process via Hot Melt Extrusion Technology (Part I). Int. J. Pharm..

[27] Alshammari N.D., Elkanayati R., Vemula S.K., Al Shawakri E., Uttreja P., Almutairi M., Repka M.A. (2024). Advancements in Colon-Targeted Drug Delivery: A Comprehensive Review on Recent Techniques with Emphasis on Hot-Melt Extrusion and 3D Printing Technologies. AAPS PharmSciTech.

[28] Srinivasan P., Almutairi M., Youssef A.A.A., Almotairy A., Bandari S., Repka M.A. (2023). Numerical Simulation of Five Different Screw Configurations Used during the Preparation of Hot-Melt Extruded Kollidon^®^ and Soluplus^®^ Based Amorphous Solid Dispersions Containing Indomethacin. J. Drug Deliv. Sci. Technol..

[29] Almotairy A., Alyahya M., Althobaiti A., Almutairi M., Bandari S., Ashour E.A., Repka M.A. (2023). Disulfiram 3D Printed Film Produced via Hot-Melt Extrusion Techniques as a Potential Anticervical Cancer Candidate. Int. J. Pharm..

[30] Uhumwangho M.U., Ramana M.K.V. (2011). In-Vitro Characterization of Optimized Multi-Unit Dosage Forms of Theophylline and Its Solid State Characterisation. J. Appl. Sci. Environ. Manag..

[31] Mokra D., Mokry J. (2021). Phosphodiesterase Inhibitors in Acute Lung Injury: What Are the Perspectives?. Int. J. Mol. Sci..

[32] Aitipamula S., Wong A.B.H., Kanaujia P. (2018). Evaluating Suspension Formulations of Theophylline Cocrystals with Artificial Sweeteners. J. Pharm. Sci..

[33] Rodríguez-Hornedo N., Lechuga-Ballesteros D., Wu H.-J. (1992). Phase Transition and Heterogeneous/Epitaxial Nucleation of Hydrated and Anhydrous Theophylline Crystals. Int. J. Pharm..

[34] https://bitterdb.agri.huji.ac.il/compound.php?mode_organism=default&id=514.

[35] Burapapadh K., Warintaksa P., Ruksapram S., Saokham P. (2024). Development of Taste-Masked Enteric Granules Containing Diclofenac Sodium Utilizing Eudragit^®^ E PO as a Taste-Masking Agent. Sci. Eng. Health Stud..

[36] Mashaqbeh H., Obaidat R., Alsmadi M.M., Athamneh T. (2024). Comparison between Solvent Evaporation and Supercritical CO_2_ Technology in Taste-Masking of Azithromycin Bitter-Taste Using PH-Sensitive Eudragit EPO or Eudragit S100 Polymers. J. Appl. Pharm. Sci..

[37] Abdelhakim H.E., Coupe A., Tuleu C., Edirisinghe M., Craig D.Q.M. (2021). Utilising Co-Axial Electrospinning as a Taste-Masking Technology for Paediatric Drug Delivery. Pharmaceutics.

[38] Keeley A., Teo M., Ali Z., Frost J., Ghimire M., Rajabi-Siahboomi A., Orlu M., Tuleu C. (2019). In Vitro Dissolution Model Can Predict the in Vivo Taste Masking Performance of Coated Multiparticulates. Mol. Pharm..

[39] https://pharma.basf.com/files/brochures/Investigation_the_impact_of_Kollicoat_formulation_concepts_taste_masking_functionality.pdf.

[40] Pradhan A. (2018). A Study on the Taste Masking Ability of Kollicoat® Mae 100-55 on Caffeine Citrate via Hot Melt Extrusion Technology. Mater’s Thesis.

[41] Nukala P.K., Palekar S., Patki M., Fu Y., Patel K. (2019). Multi-Dose Oral Abuse Deterrent Formulation of Loperamide Using Hot Melt Extrusion. Int. J. Pharm..

[42] Goyanes A., Kobayashi M., Martínez-Pacheco R., Gaisford S., Basit A.W. (2016). Fused-Filament 3D Printing of Drug Products: Microstructure Analysis and Drug Release Characteristics of PVA-Based Caplets. Int. J. Pharm..

[43] Tung N.T., Tran C.S., Nguyen T.L., Hoang T., Trinh T.D., Nguyen T.N. (2018). Formulation and Biopharmaceutical Evaluation of Bitter Taste Masking Microparticles Containing Azithromycin Loaded in Dispersible Tablets. Eur. J. Pharm. Biopharm..

[44] Muhindo D., Ashour E.A., Almutairi M., Repka M.A. (2022). Development and Evaluation of Raloxifene Hydrochloride-Loaded Subdermal Implants Using Hot-Melt Extrusion Technology. Int. J. Pharm..

[45] Ramos P. (2022). Application of Thermal Analysis to Evaluate Pharmaceutical Preparations Containing Theophylline. Pharmaceuticals.

[46] Nyamba I., Jennotte O., Sombié C.B., Lechanteur A., Sacre P.Y., Djandé A., Semdé R., Evrard B. (2023). Preformulation Study for the Selection of a Suitable Polymer for the Development of Ellagic Acid-Based Solid Dispersion Using Hot-Melt Extrusion. Int. J. Pharm..

[47] Sakkal M., Arafat M., Yuvaraju P., Beiram R., Ali L., Altarawneh M., Hajamohideen A.R., AbuRuz S. (2024). Effect of Hydration Forms and Polymer Grades on Theophylline Controlled-Release Tablet: An Assessment and Evaluation. Pharmaceuticals.

[48] Rokhade A.P., Shelke N.B., Patil S.A., Aminabhavi T.M. (2007). Novel Interpenetrating Polymer Network Microspheres of Chitosan and Methylcellulose for Controlled Release of Theophylline. Carbohydr. Polym..

[49] Georgieva Y., Kassarova M., Kokova V., Apostolova E., Pilicheva B. (2020). Taste Masking of Enalapril Maleate by Microencapsulation in Eudragit EPO^®^ Microparticles. Pharmazie.

[50] Pein M., Preis M., Eckert C., Kiene F.E. (2014). Taste-Masking Assessment of Solid Oral Dosage Forms—A Critical Review. Int. J. Pharm..

[51] Legin A., Rudnitskaya A., Clapham D., Seleznev B., Lord K., Vlasov Y. (2004). Electronic Tongue for Pharmaceutical Analytics: Quantification of Tastes and Masking Effects. Anal. Bioanal. Chem..

[52] Petrovick G.F., Breitkreutz J., Pein-Hackelbusch M. (2016). Taste-Masking Properties of Solid Lipid Based Micropellets Obtained by Cold Extrusion-Spheronization. Int. J. Pharm..

[53] Uchida T. (2024). Taste Sensor Assessment of Bitterness in Medicines: Overview and Recent Topics. Sensors.

[54] Alshetaili A.S., Almutairy B.K., Tiwari R.V., Morott J.T., Alshehri S.M., Feng X., Alsulays B.B., Park J.-B., Zhang F., Repka M.A. (2016). Preparation and Evaluation of Hot-Melt Extruded Patient-Centric Ketoprofen Mini-Tablets. Curr. Drug Deliv..

[55] (2017). SPI Pharma, Actimask®: Taste Masked Actives. https://www.spipharma.com/media/2860/actimask-psb-oct-2017.pdf.

[56] Karagianni A., Kachrimanis K., Nikolakakis I. (2018). Co-Amorphous Solid Dispersions for Solubility and Absorption Improvement of Drugs: Composition, Preparation, Characterization and Formulations for Oral Delivery. Pharmaceutics.

[57] Patel N.G., Banella S., Serajuddin A.T.M. (2023). Moisture Sorption by Polymeric Excipients Commonly Used in Amorphous Solid Dispersions and Its Effect on Glass Transition Temperature: III. Methacrylic Acid-Methyl Methacrylate and Related Copolymers (Eudragit^®^). Int. J. Pharm..

[58] Bhujbal S.V., Mitra B., Jain U., Gong Y., Agrawal A., Karki S., Taylor L.S., Kumar S., Zhou Q.T. (2021). Pharmaceutical Amorphous Solid Dispersion: A Review of Manufacturing Strategies. Acta Pharm. Sin. B.

[59] Buckley L.A., Salunke S., Thompson K., Baer G., Fegley D., Turner M.A. (2018). Challenges and strategies to facilitate formulation development of pediatric drug products: Safety qualification of excipients. Int. J. Pharm..

[60] Saito J., Agrawal A., Patravale V., Pandya A., Orubu S., Zhao M., Andrews G.P., Petit-Turcotte C., Landry H., Croker A. (2022). The Current States, Challenges, Ongoing Efforts, and Future Perspectives of Pharmaceutical Excipients in Pediatric Patients in Each Country and Region. Children.

