# Supplementary material for: Kollicoat® Smartseal 100P for Developing Theophylline Pellets: Exploring Taste-Masking Potential for Pediatric Applications
Source: Pharmaceutics. 2025 Mar 25;17(4):413. doi: 10.3390/pharmaceutics17040413 (PMC12030470; doi:10.3390/pharmaceutics17040413)
Supplement: Supplementary file 1 [file pharmaceutics-17-00413-s001.zip › pharmaceutics-3538953-supplementary.pdf]

Article

Kollicoat® Smartseal 100P for developing Theophylline Pellets: Exploring Taste-Masking Potential for Pediatric Applications

Neeraja Komanduri [a], Mashan Almutairi [b], Rasha M. Elkanayati [a], Nagireddy Dumpa [a], Arun Butreddy [a], Suresh Bandari [a], Michael A. Repka [a,c]

[a] Department of Pharmaceutics and Drug Delivery, School of Pharmacy, The University of Mississippi, University, MS, 38677, USA

[b] Department of Pharmaceutics, College of Pharmacy, University of Hail, Hail 81442, Saudi Arabia

[c] Pii Center for Pharmaceutical Technology, The University of Mississippi, University, MS 38677, USA

* Correspondence: marepka@olemiss.edu; Tel.: +1 662 915 1155

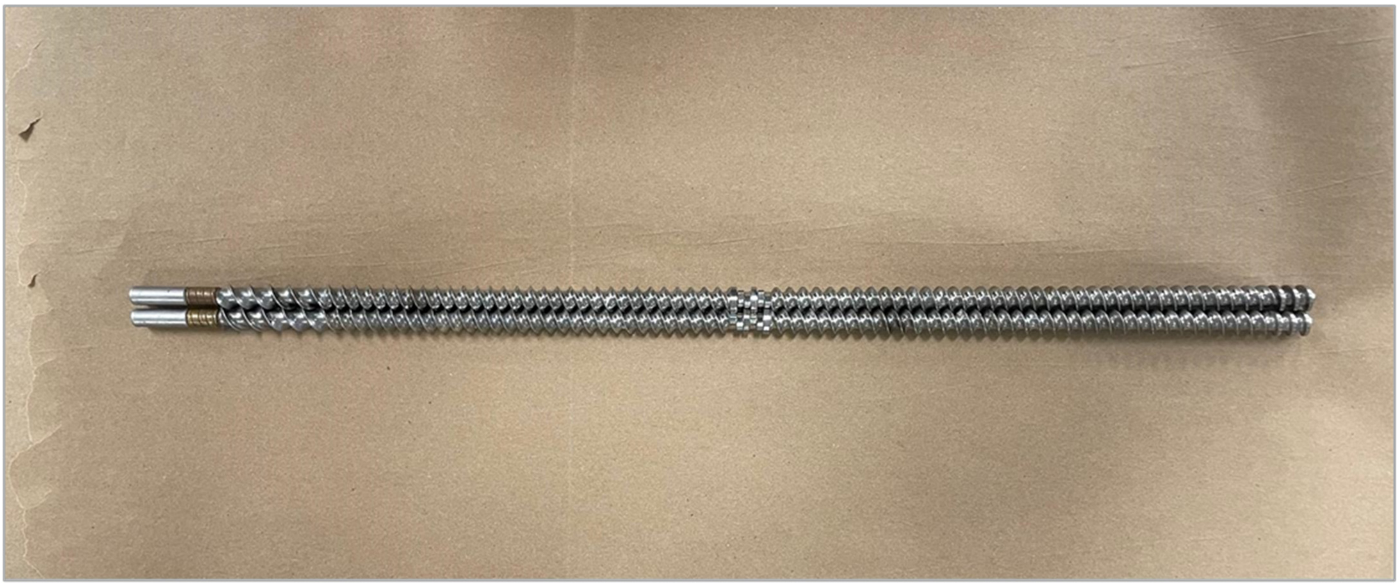

Figure S1. Screw configuration employed for extruding the formulations

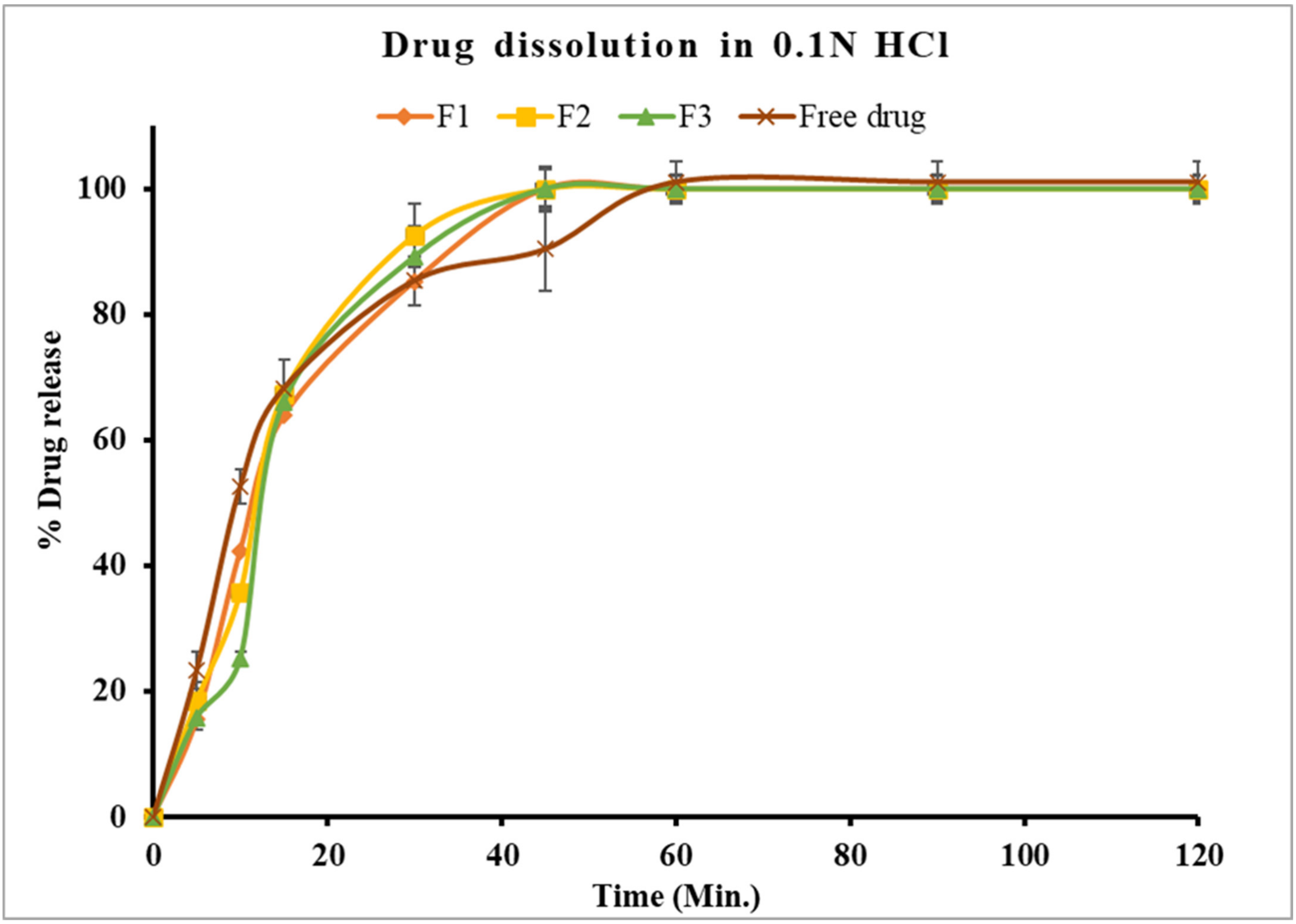

Figure S2. Drug dissolution of theophylline and Kollicoat Smartseal 100P based formulations containing 30% PEG 1500 (F1, F2, F3) in 0.1 N HCl

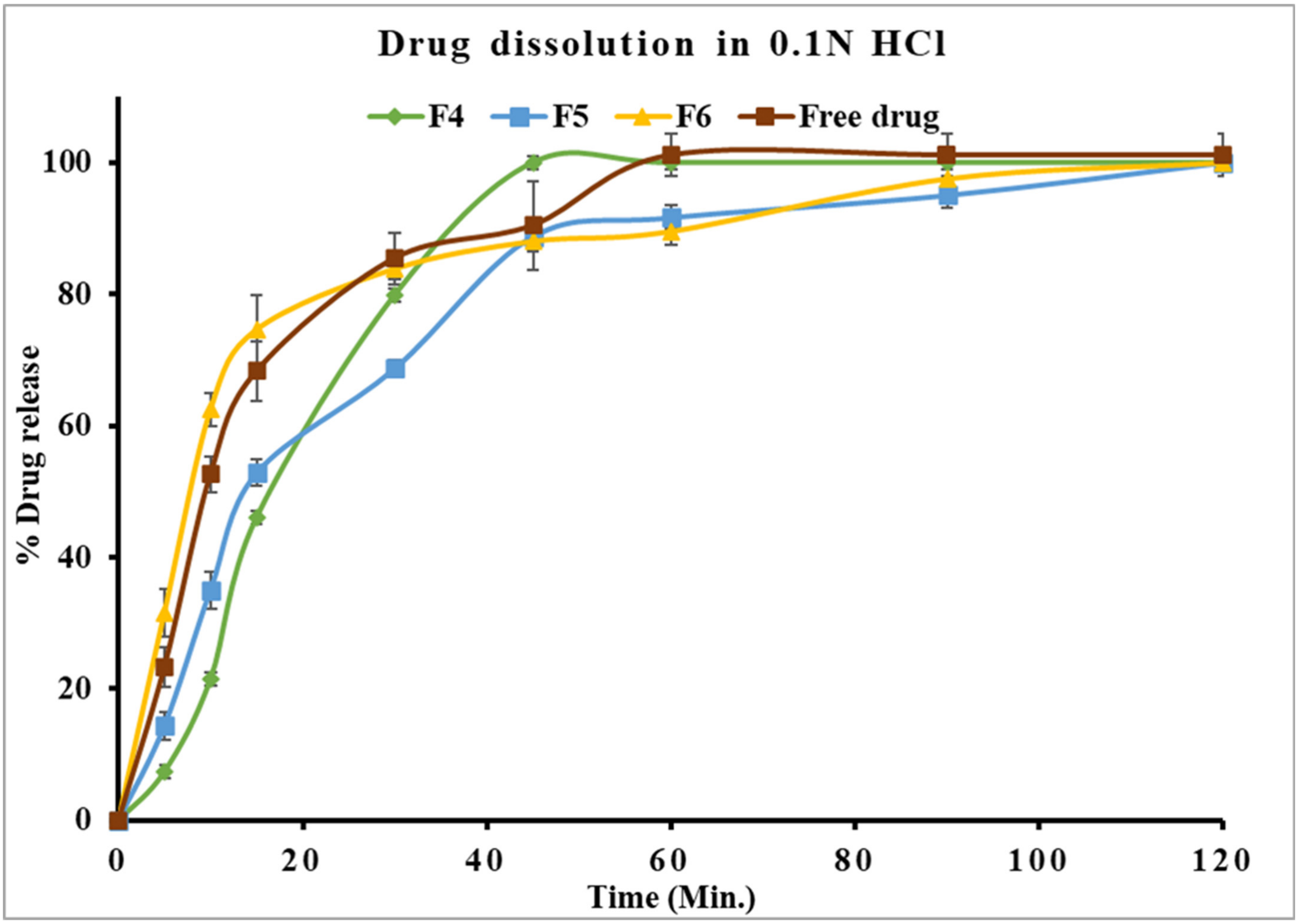

Figure S3. Drug dissolution of theophylline and Kollicoat Smartseal 100P based formulations containing 20% PEG 1500 (F4, F5, F6) in 0.1 N HCl
